# A Novel Role of Spred2 in the Colonic Epithelial Cell Homeostasis and Inflammation

**DOI:** 10.1038/srep37531

**Published:** 2016-11-21

**Authors:** Sakuma Takahashi, Teizo Yoshimura, Takahiro Ohkura, Masayoshi Fujisawa, Soichiro Fushimi, Toshihiro Ito, Junya Itakura, Sakiko Hiraoka, Hiroyuki Okada, Kazuhide Yamamoto, Akihiro Matsukawa

**Affiliations:** 1 Department of Gastroenterology and Hepatology, Okayama University Graduate School of Medicine, Dentistry and Pharmaceutical Sciences, Okayama, 700-8558, Japan; 2 Department of Pathology and Experimental Medicine, Okayama University Graduate School of Medicine, Dentistry and Pharmaceutical Sciences, Okayama, 700-8558, Japan; 3 Department of Immunology, Nara Medical University, Nara, 634-8521, Japan

## Abstract

Rapid and adequate mucosal healing is important for a remission of ulcerative colitis (UC) patients. Here, we examined whether Spred2, a member of the Sprouty-related EVH1-domain-containing proteins that inhibit the Ras/Raf/ERK pathway, plays a role in colonic mucosal homeostasis and inflammation by using Spred2 knockout (KO) mice. We first detected increased epithelial cell proliferation and cadherin 1 expression in the colon of naïve Spred2 KO mice compared to wild-type mice. Interestingly, Spred2 KO mice were resistant to dextran sulfate sodium (DSS)-induced acute colitis as indicated by lower levels of body weight loss and disease activity index. Histologically, epithelial cell injury and inflammation were milder in the colonic mucosa of Spred2 KO mice on day 3 and almost undetectable by day 8. Experiments with bone chimeric mice indicated that Spred2-deficiency in non-hematopoietic cells was responsible for the reduced sensitivity to DSS. Finally, Spred2 KO mice developed significantly fewer tumors in response to azoxymethane plus DSS. Taken together, our results demonstrate, for the first time, that Spred2 plays an important role in the regulation of colonic epithelial cell proliferation and inflammation by potentially down-regulating the activation of ERK. Thus, Spred2 may be a new therapeutic target for the treatment of UC.

Ulcerative colitis (UC), one of the two major forms of the inflammatory bowel disease (IBD), is a chronic disease characterized by strong inflammatory responses and ulceration in the colon mucosa^1^. The incidence and prevalence of UC are increasing with time in many regions around the world^2^. The etiology of UC is believed to involve inappropriate host responses to the complex commensal microbial flora in the gut^3^,^4^,^5^. Intestinal barrier dysfunction is a main feature of UC^6^,^7^. Clinically, rapid and adequate mucosal healing after treatment is an important indicator for a long-term remission in UC patients and a goal of treatments^8^,^9^,^10^. However, achieving mucosal healing is difficult in some patients despite the latest advance in the treatments.

The intestinal epithelium is a single cell layer that forms the only barrier between the host and the luminal gastrointestinal contents. Maintenance of this cell layer, which turns over in every 3–5 days, is dependent on the tight regulation of cell proliferation and apoptosis^11^,^12^,^13^. The maintenance of intestinal homeostasis is regulated by the coordinated activation of signaling molecules. Various molecules, such as growth factors, inflammatory cytokines and toll-like receptor ligands, stimulate intestinal epithelial cell growth, proliferation, and differentiation^14^,^15^,^16^,^17^,^18^ via binding to their cognate transmembrane receptors. Thus, identifying factors that affect mucosal healing can greatly contribute to the advance of UC treatment.

Receptor tyrosine kinases transmit signals that regulate multiple cellular processes, including proliferation, differentiation, migration, survival and metabolism^19^,^20^. Activated receptors utilize signaling pathways, such as the Ras/Raf/ERK (extracellular signal-regulated kinase), the PI3K (phosphoinositide 3-kinase)-Akt, and the Signal Transducer and Activator of Transcription (STAT) pathways^21^,^22^,^23^. Epidermal growth factor (EGF) is one of the major growth-stimulating factors of intestinal epithelial cells^24^,^25^,^26^. A randomized, double-blind clinical trial of EGF enemas using patients with active left-sided UC indicated that EGF enemas were effective and caused no colonic dysplasia^27^. Hepatocyte growth factor (HGF) is also a major growth-stimulating factor^28^. Intraperitoneal administration of HGF facilitated colonic mucosal repair in a rat experimental colitis model^29^. EGF and HGF bind to their cognate receptor EGFR and c-MET, respectively^30^,^31^. A crosstalk between EGFR and c-MET can enhance epithelial cell proliferation and wound healing through the Ras/Raf/ERK and the PI3K-Akt pathways^32^,^33^,^34^.

Sprouty-related enabled/vasodilator-stimulated phosphoprotein homology 1 domain-containing protein (Spred)1, 2 and 3 are a family of proteins containing a cysteine-rich domain related to Sprouty of *Drosophila* at the carboxy terminus and inhibit, similar to Sprouty, the activation of MAP kinase by suppressing the phosphorylation and activation of Raf 35,36. Spred1 and Spred3 are preferentially expressed in the brain and cerebellum, whereas Spred2 is ubiquitously expressed in various tissues, including the colon^37^,^38^. Interestingly, mutations in the Spred1 gene are implicated in the pathogenesis of Legius syndrome, an autosomal dominant human disorder that resembles neurofibromatosis-1^39^ and pediatric acute myeloblastic leukemia^40^. The results of genome-wide association studies suggest the Spred2 gene as a candidate gene on IBD susceptibility loci^41^; however, the physiological role of Spred2 remains unknown. Here, we demonstrate, for the first time, that Spred2 plays an important role in the regulation of mucosal epithelial cell homeostasis and inflammation in the colon.

## Results

### The proliferation of colonic epithelial cells and the expression of the *cadherin 1* gene are increased in the colon of naïve Spred2 knockout (KO) mice

**Figure 1:**
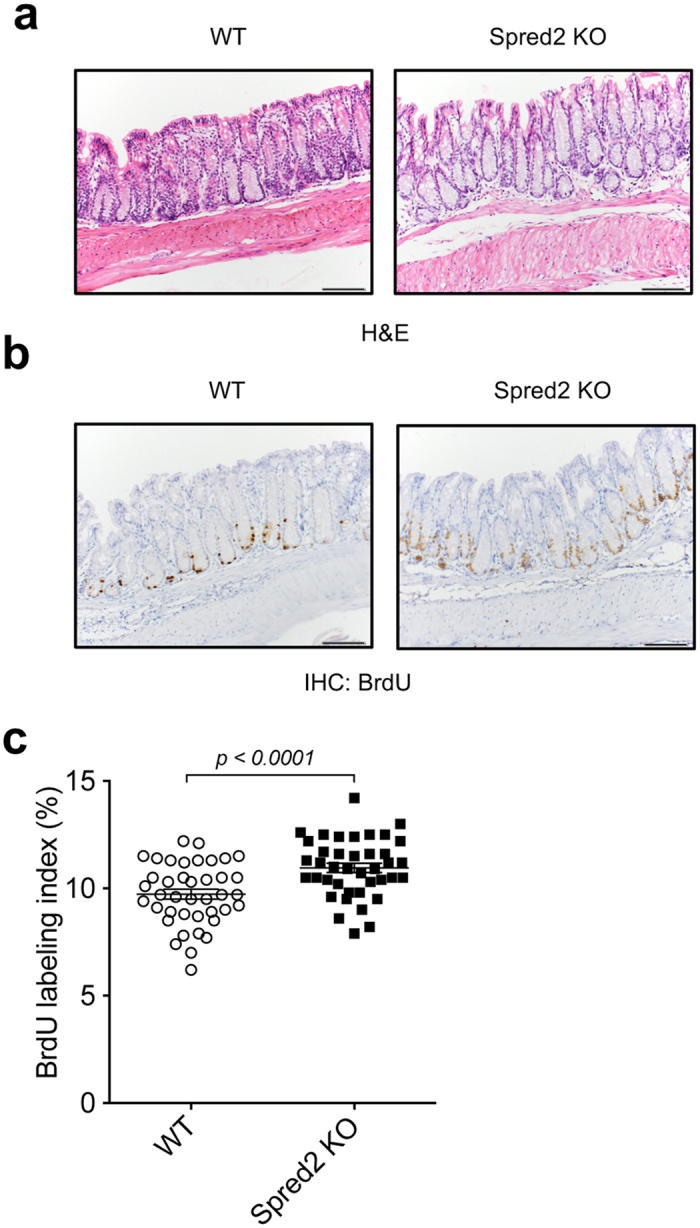
The incorporation of BrdU in the colon of naïve WT and Spred2 KO mice. Colons were harvested from naïve WT or Spred2 KO mice 2 hours after intra-peritoneal injection of BrdU (50 μg/g body weight). (**a**) Photos of H&E staining of the distal portion of colon tissues from untreated WT or Spred2 KO mice are shown. (**b**) The incorporation of BrdU was examined by immunohistochemistry. Representative results from four animals are shown (original scale bar, 100 μm). (**c**) The incorporation of BrdU in colonic epithelial cells of WT and Spred2 KO mice was quantitated and presented as BrdU labeling index. Data is presented as the mean ± SEM. ***p < *0.0001*. n = 4.

To examine the role of Spred2 in the colonic epithelial cell homeostasis and inflammation, we first examined the colon of naïve wild-type (WT) and Spred2 KO mice. Histologically, there was no notable difference between the colon of naïve WT and Spred2 KO mice by H&E staining, including the crypt height (Fig. 1a). However, interestingly, the incorporation of 5-Bromo-2′-deoxyuridine (BrdU) was significantly higher in the epithelial cells of Spred2 KO mice than those of WT mice (Fig. 1b,c), suggesting that Spred2 down-regulates the proliferation of colonic epithelial cells *in vivo*.

**Figure 2:**
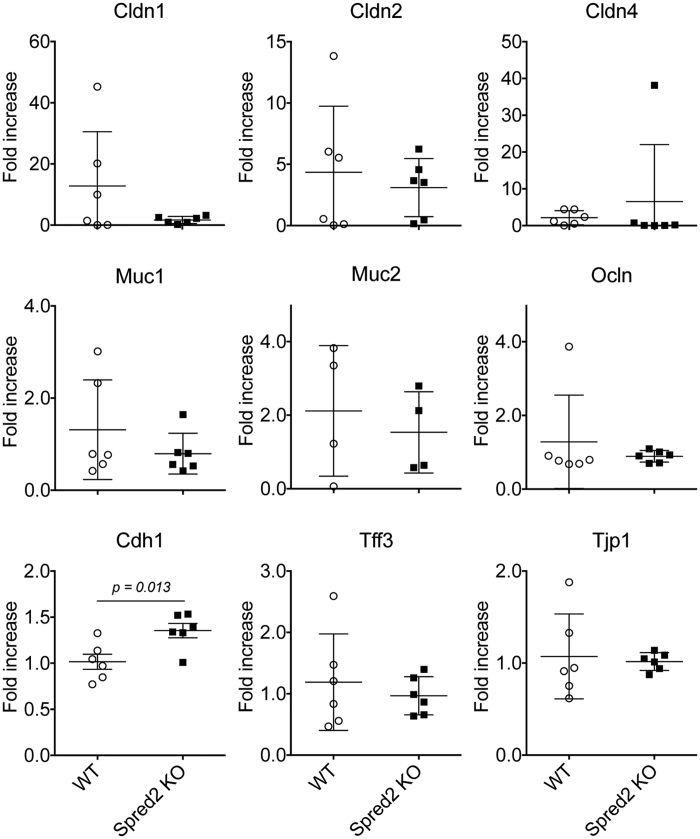
The expression of genes associated with mucosal barrier functions. The expression of nine genes, including *Cldn 1,2* and *4*, *Muc1* and *2*, *Ocln*, *Cdh 1*, *Tff3* and *Tjp1* genes, in the colon of naïve WT or Spred2 KO mice was examined by qRT-PCR. Data is presented as the mean ± SEM. n = 4 for Muc2, n = 6 for the other 8 genes.

We next evaluated the effect of Spred2-deficiency on the integrity of the epithelial barrier functions by examining the expression of nine genes whose products are involved in tight junction formation, including the *claudin (Cldn) 1, 2* and *4*, *mucin (Muc) 1* and *2, occludin (Ocln), trefoil factor 3 (Tff3), cadherin (Cdh) 1* and *tight junction protein (Tjp) 1* gene^14^,^42^,^43^,^44^, by qRT-PCR. Among the genes, the expression of *Cdh1*, but not others, was significantly higher in the colon of naïve Spred2 KO mice than that of naïve WT mice (Fig. 2), suggesting that Spred2 may regulate the epithelial barrier functions in mice.

### Spred2 KO mice are resistant to dextran sulfate sodium (DSS)-induced acute colitis

**Figure 3:**
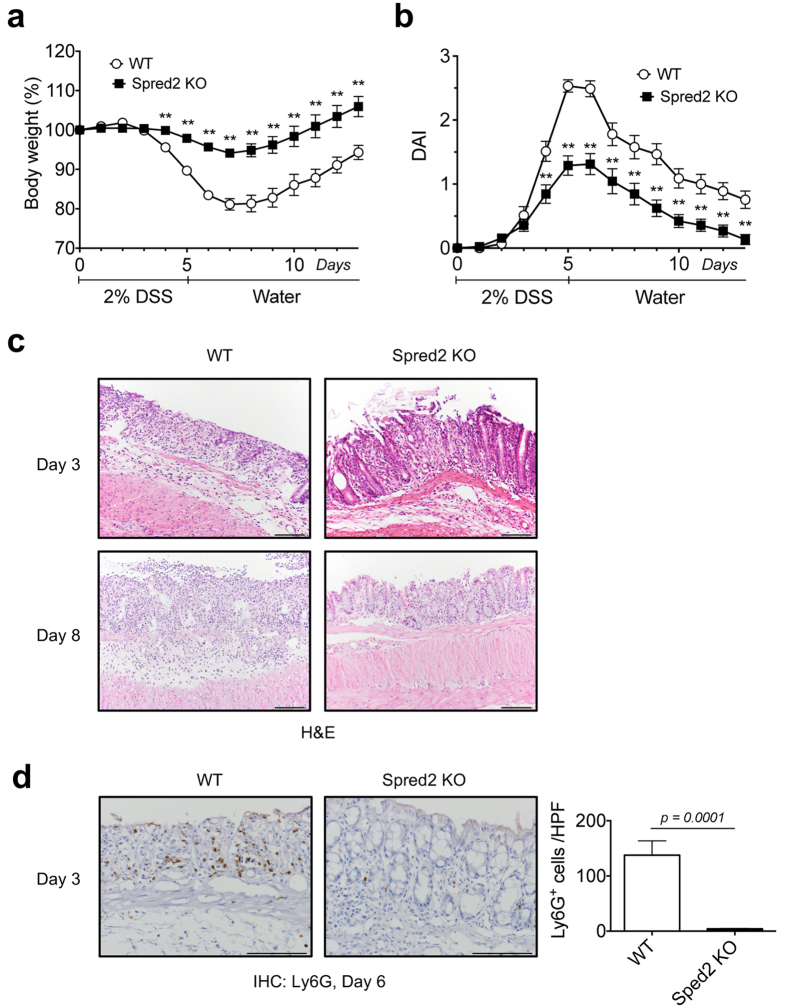
Spred2 KO mice are protected from DSS-induced acute colitis. WT and Spred2 KO mice were given 2% of DSS in drinking water for 5 days, followed by regular water. Changes in body weight (**a**) and DAI (**b**) were examined. Data is presented as the mean ± SEM. **p < *0.01*. n = 15. (**c**) Colons were harvested from mice on day 3 or 8 and processed for H&E staining. Representative results from five mice are shown (original scale bar, 100 μm). (**d**) The infiltration of Ly6G-positive cells (neutrophils) on day 6 was evaluated by immunohistochemistry. The number of positive cells was counted and presented as the mean ± SEM per HPF. n = 3 for WT and n = 4 for Spred2 KO mice.

DSS-induced colitis is one of the most frequently used rodent models of UC^45^,^46^. It is widely accepted that DSS is toxic to colonic epithelial cells and DSS-induced breakdown of mucosal epithelial barrier function allows the entry of luminal antigens and microorganisms into the mucosa resulting in overwhelming inflammatory response^47^. To evaluate the role of Spred2 in colonic inflammation and tissue repair, WT and Spred2 KO mice were given 2% DSS in drinking water ad libitum, followed by water. As shown in Fig. 3a, WT mice began to lose weight on day 4, continued to lose weight until day 7, and slowly recovered thereafter. Disease activity index (DAI) also increased on day 4, peaked on day 5 and 6 and then gradually returned to the original level (Fig. 3b). In contrast to WT mice, Spred2 KO mice showed only a minor body weight loss and a minor increase in DAI (Fig. 3a,b), suggesting that Spred2 KO mice are resistant to DSS-induced tissue injury.

Histologically, a significant level of epithelial cell damage and inflammatory cell infiltration was detected in the distal region of WT colon on day 3 and they became more severe by day 8 by H&E staining. In contrast, the colon of Spred2 KO mice showed only a low level of epithelial cell damage and inflammatory response on day 3 and returned to almost normal by day 8 (Fig. 3c). The infiltration of Ly6G-positive cells, most likely neutrophils, was markedly lower in the colon of Spred2 KO mice (Fig. 3d). Thus, Spred2 KO mice are resistant to DSS-induced tissue injury and inflammation.

### Absence of Spred2 in non-hematopoietic cells attenuated DSS-induced colitis

**Figure 4:**
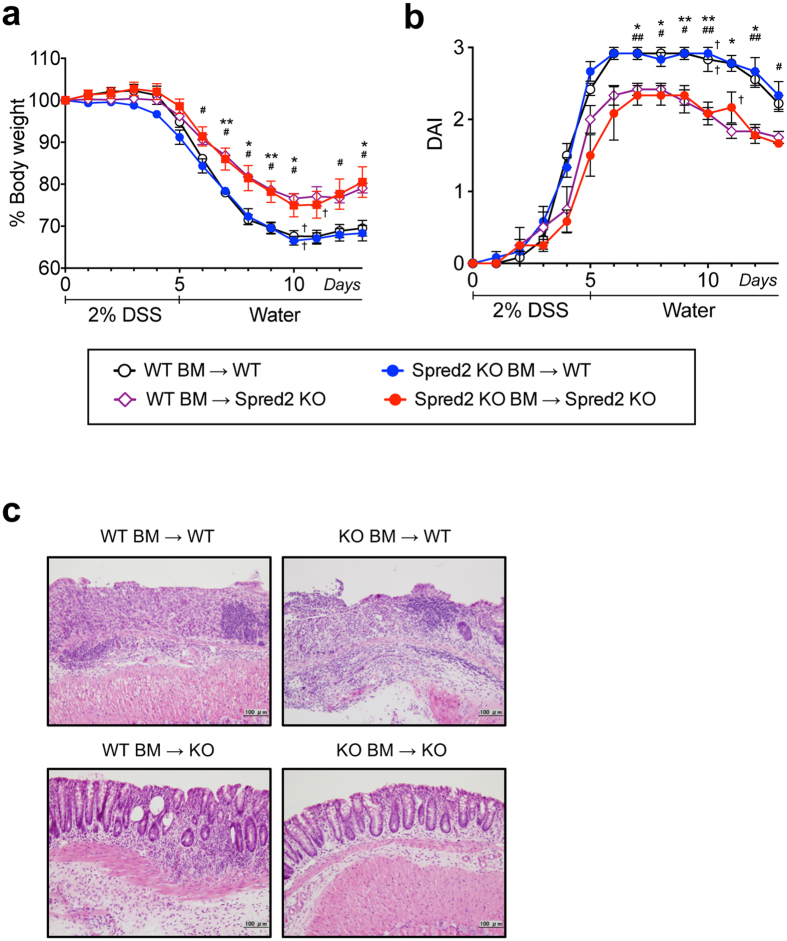
DSS-induced acute colitis in BM chimeric mice. BM chimeric mice between WT and Spred2 KO mice were produced as described in the M&M section. Mice were given 2% DSS in drinking water for 5 days, followed by regular water. (**a**) Changes in body weight were examined. Data is presented as the mean ± SEM. *p < *0.05*, **p < *0.01*. WT BM → WT vs WT BM → Spred2 KO. ^#^*p* < *0.05*, **p < *0.01*. Spred2 KO BM → WT vs Spred2 KO BM → Spred2 KO (n = 4). The cross indicates death to a mouse. (**b**) Disease activity index (DAI) was calculated as described in the M&M section. Data is presented as the mean ± SEM. *p < *0.05*, **p < *0.01*. WT BM → WT vs WT BM → Spred2 KO. ^#^*p* < *0.05*, ^##^*p* < *0.01*. Spred2 KO BM → WT vs Spred2 KO BM → Spred2 KO (n = 4). The cross indicates death to a mouse. (**c**) Colons were harvested on day 13 and processed for H&E staining. Representative results from four animals are shown (original scale bar, 100 μm).

As described above, Spred2 is ubiquitously expressed in various tissues^37^,^38^. To determine whether hematopoietic or non-hematopoietic Spred2 is associated with the decreased DSS sensitivity, we generated bone marrow (BM) chimeric mice. As shown in Fig. 4a and b, Spred2 KO mice which received Spred2 KO BM cells (KO BM → KO) lost less body weight than WT mice which received WT BM cells (WT BM → WT), and showed a lower DAI, consistent with our results with non-chimeric mice presented in Fig. 3. Transplantation of Spred2 KO BM cells into WT mice (KO BM → WT) or WT BM cells into Spred2 KO mice (WT BM → KO) did not alter the body weight loss or DAI observed in WT or Spred2 KO mice. By H&E staining, the presence of severe inflammatory responses and tissue damage were detected in WT mice that received WT BM cells or Spred2 KO BM cells but not in Spred2 KO mice that received WT BM cells or Spred2 KO BM cells on day 13 (Fig. 4c). These results indicated that the loss of Spred2 in non-hematopoietic cells, but not in hematopoietic cells, was responsible for the attenuated DSS-induced colitis detected in systemic Spred2 KO mice.

### Spred2 KO mice develop significantly fewer tumors than WT mice in an azoxymethane (AOM)/DSS model

**Figure 5:**
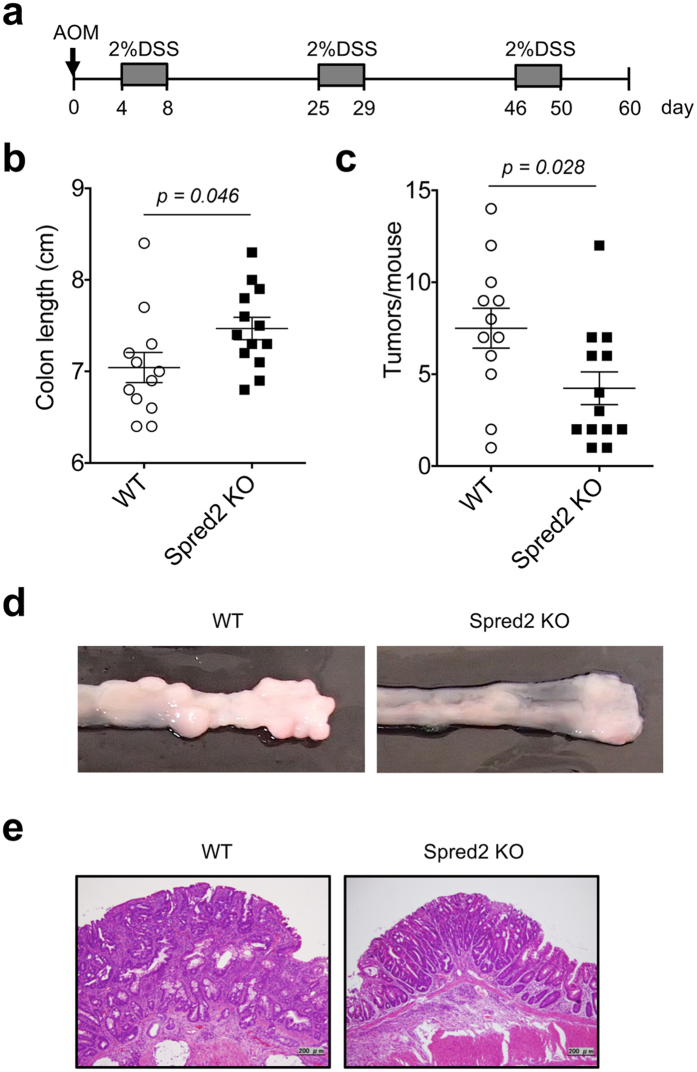
Tumor formation in WT and Spred2 KO mouse colon after treatment with AOM plus DSS. (**a**) The experimental scheme of the colitis-associated cancer model used in the study. (**b**) Colons were harvested on day 60 from WT and Spred2 KO mice and colon length was measured. Data is presented as the mean ± SEM (n = 12 for WT mice and n = 13 for Spred2 KO mice). (**c**) The number of tumors whose diameter was more than 1 mm was counted. Data is presented as the mean ± SEM (n = 12 for WT mice and n = 13 for Spred2 KO mice). (**d**) Representative photos of tumors that arose in WT or Spred2 KO mice. (**e**) Photos of typical tumors developed in WT or Spred2 KO mice. H&E staining. The original scale bar was 200 μm.

The AOM/DSS-induced colon cancer model is one of the most frequently used rodent colitis-associated cancer model^48^. To examine the role of Spred2 in the development of colitis-associated colon cancer, mice were treated with AOM, followed by three cycles of 2% DSS treatment and recovery (Fig. 5a). Sixty days after AOM injection, all mice were euthanized and the length of each colon and the number of tumors were examined. The length of colons of WT mice was significantly shorter than those of Spred2 KO mice (Fig. 5b). Furthermore, WT mice developed significantly higher number of tumors than Spred2 KO mice (7.5 vs 4.2 per colon) (Fig. 5c,d). The growth of epithelial cells was more prominent in tumors of WT mice than those of Spred2 KO mice (Fig. 5e) and tumors in the colon of WT mice appear to be larger than those of Spred2 KO mice. These results are likely due to more severe inflammatory responses in the colon of WT mice than Spred2 KO mice.

## Discussion

Under normal circumstances, IECs are constantly shed from the tips of villi after cell death. To maintain intestinal homeostasis, controlled proliferation of IECs is required to prevent loss of epithelial barrier function^10^. In this study, we examined a biological role of Spred2 in colonic epithelial homeostasis and inflammation using Spred2 KO mice. We found that the proliferation of epithelial cells and the expression of the *Cdh1* gene were significantly increased in the colon of Spred2 KO mice compared to WT mice in a naïve state. Interestingly, Spred2 KO mice were less sensitive to DSS-induced acute colitis than WT mice and developed a fewer number of tumors in a chemically induced colon cancer level. Thus, we, for the first time, identified Spred2 as an important molecule that regulates the proliferation of colon epithelial cells and inflammation.

Previous studies have shown that the activation of the Ras/Raf/ERK pathway enhances colonic mucosal repair. Heparin-binding epidermal growth factor-like growth factor enhanced intestinal restitution after intestinal ischemia/reperfusion via PI3K/Akt and Ras/Raf/ERK activation^49^. Raf was shown to protect the mouse colon against epithelial injury and inflammation, and promote recovery from acute DSS-induced colitis by both Ras/Raf/ERK-dependent and -independent pathways^50^. Rebamipide, an amino acid derivative of 2(1 H)-quinoline used as a gastric mucosal protective and ulcer-healing agent, enhanced the migration of intestinal epithelial cells via Ras/Raf/ERK activation in a rat acute colitis model induced with trinitrobenzene sulfonic acid^51^. Oral administration of insulin stimulated intestinal epithelial cell turnover following massive small bowel resection in a rat and a cell culture model via PI3K/Akt and Ras/Raf/ERK activation^52^. Inhibition of Ras/Raf/ERK by U0126 significantly decreased cell proliferation and migration of rat intestinal epithelial cell line IEC-6 and worsened intestinal ischemia/reperfusion injury^53^. As described above, Spred proteins are negative feedback regulators of the ERK/MAPK pathway^35^, and Spred2 had the capacity to suppress VEGFR-3-mediated ERK activation in VEGFR-3-transfected 293 T cells^54^. Thus, Spred2 may play an important role in the proliferation and migration of colonic epithelial cells by regulating the activation of ERK.

In the present study, we demonstrated that Spred2 KO mice were resistant to DSS-induced acute colitis; however, it remains unclear how Spred2-deficincy results in the resistance to DSS-induced colitis. The incorporation of BrdU in the colonic epithelia and the expression of the *Cdh1* gene in the colon tissue were significantly increased in Spred2 KO mice compared to WT mice, suggesting that increased epithelial restitution and barrier functions in Spred2 mice may account for our observation. The Raf/MEK/ERK pathway also regulates cell survival by controlling the activity or abundance of the members of the Bcl-protein family to promote cell survival^55^. Therefore, Spred2-deficiency might increase the survival of colonic epithelial cells, which could lead to the resistance to DSS-induced acute colitis.

The Ras/Raf/ERK pathway is shown to influence not only epithelial cells but also immune cells, such as T cells and macrophages. Pharmacologic inhibition of Ras/Raf/ERK was previously shown to enhance *in vitro* Th17 differentiation and increase the gene expression of *il-17a*, *il-17f*, *il-21*, *il-22*, and *il-23r*^56^. Th17 cells are now known to regulate the pathogenic mechanism of inflammatory bowel disease^57^,^58^. Intestinal microbiota also plays a critical role in the regulation of intestinal epithelial cell turnover and promotion of epithelial restitution^43^. Particularly, in DSS-induced colitis, bacteria that translocated to the mucosa interact with mucosal cells, such as macrophages, via toll-like receptors and promote the production of pro-inflammatory cytokines, resulting in overwhelming inflammatory responses^48^. We previously reported that Spred2-deficiency increased the production of pro-inflammatory cytokines by LPS-activated alveolar macrophages, and exacerbated LPS-induced lung injury^59^. In this study, however, the inflammatory response in the colonic mucosa of Spred2 mice was milder than WT mice, and only a small number of leukocytes infiltrated the mucosa of Spred2 KO mice; thus, the effect of Spred2-deficiency on macrophages cannot explain the decreased inflammatory responses detected in the colon of Spred2 KO mice. Furthermore, Spred2-deficiency in non-hematopoietic cells, but not hematopoietic cell, was responsible for the DSS-resistance in this study; therefore, Spred2 expressed in non-hematopoietic cells, such as epithelial cells, must be playing a critical role. Additional studies are necessary to identify the exact mechanism whereby Spred2-deficiency protects the colon from DSS-induced insults.

The Ras/Raf/ERK and the PI3K/Akt pathway also influence colon cancer growth^60^, suggesting that Spred2-deficiency may increase tumor development. However, Spred2 KO mice developed significantly fewer tumors than WT mice. Severity of colon inflammation is a risk factor for colorectal neoplasia in ulcerative colitis^61^,^62^. HGF ameliorates mucosal injuries leading to inhibition of colon cancer development in mice^63^. These reports suggest that chronic mucosal inflammation promotes colitis associated colon cancer growth. In Spred2 KO mice, the degree of the inflammatory response after DSS-treatment was clearly less severe than WT mice, providing the reason why fewer tumors developed in Spred2 KO mice in response to AOM/DSS-treatment.

To analyze the role of Spred2 expressed in colonic epithelial cells, we knocked down Spred2 expression in the human Caco-2 colon cancer cell line using siRNA and evaluated its effects *in vitro*. Interestingly, we detected a significant increase in the cell proliferation, migration and ERK phosphorylation in siRNA-transfected Caco-2 cells compared to non-transfected cells. However, the increase was very modest despite that the expression of Spred2 mRNA expression was reduced by 80%. Unexpectedly, about 50% of Spred2 protein still remained in the siRNA-transfected cells, potentially resulting in only small changes (Supplementary Figure 1 and 2). Thus, it will be necessary to use Spred2 knockout cells to obtain more definitive answers as to whether Spred2 regulates the proliferation and migration of colonic epithelial cells via the Ras/Raf/ERK pathway.

Rapid and adequate mucosal healing is one of the important prognostic factors for the long-term remission in UC patients. Our results obtained using Spred2 KO mice suggest that inhibition of Spred2 may be useful to prevent severe colitis and subsequent colon cancer; thus, Spred2 may be a new therapeutic target for the treatment of UC. Further investigation is needed to elucidate the mechanisms whereby Spred2 plays a role in colonic homeostasis, inflammatory responses and carcinogenesis.

## Methods

### Mice

The production of Spred2 KO mice was previously reported^54^,^64^. C57BL/6J mice were used as control. These mice were bred at the Department of Animal Resources, Okayama University, Okayama, Japan. Seven to 9-weeks old male mice were used in this study under a specific pathogen-free condition. Mice (5 per cage) were housed in a temperature-controlled environment and allowed free access to water and food. All animal protocols were approved by the Animal Care and Use Committee of the Okayama University, and all experiments were performed in accordance with relevant guidelines and regulations.

### DSS-induced colitis

DSS (MW 36,000–50,000) was purchased from MP Biomedicals (Santa Ana, CA, USA). Spred2 KO mice and WT mice were administered 2% DSS in drinking water ad libitum followed by tap water. Mice were monitored every day and DAI was calculated as described before^65^. The values were based on the weight loss percentage (0 = <1%, 1 = 1–4.99%, 2 = 5–10%, and 3 = >10%), bleeding (0 = none, 1 = small spots of blood in stool: dry anal region, 2 = large spots of blood in stool: blood appears through anal orifice, and 3 = deep red stool: blood spreads largely around the anus) and stool consistency (0 = normal stools, 1 = soft pellets not adhering to the anus, 2 = very soft pellets adhering to the anus, and 3 = liquid stool on long streams: wet anus). The average of these three values constituted the DAI. Mice were euthanized at the indicated time intervals and colons were removed for macroscopic inspection and histological analysis.

### *In vivo* BrdU labeling

BrdU (Sigma-Aldrich) was dissolved in PBS at 5 mg/ml. Mice were injected with the BrdU solution intraperitoneally at 50 μg/g body weight two hours before sacrifice. Colons were resected and fixed overnight with 10% buffered formalin and embedded in paraffin. Four-micrometer sections were cut and incubated with 1.25 μg/ml of rat anti-BrdU antibody (GeneTex, Inc., San Antonio, TX) overnight at 4 °C, followed by incubation with Histofine Simple Stain Mouse MAX PO (Rat) (Nacalai Tesque, Kyoto, Japan) for 45 minutes. Antigen was visualized with 3, 3′-diaminobenzidine substrate (Sigma-Aldrich). BrdU-labeling index was calculated as the percentage of BrdU^−^positive cells in 20 well-oriented crypts in 10 high power fields per colon section.

### Bone marrow chimeras

To make BM chimeras, recipient WT C57BL/6 mice and Spred2 KO mice (5–7 week old) were irradiated at 11 Gy (divided into two irradiations at an interval of 2 hours). Donor BM was isolated from femurs and tibias of WT and Spred2 KO mice, and 2 × 10^6^ BM cells in 100 μl RPMI 1640 were injected into recipient mice via tail vein. The following BM chimeras were created (donor → host): WT → WT, Spred2 KO → WT, Spred2 KO → Spred2 KO, and WT → Spred2 KO. Six weeks after BM reconstitution, 2% DSS in drinking water was administered to induce colitis. Mice were euthanized on day 13 and colons were dissected for macroscopic inspection and histological analysis.

### Experimental models of colitis-associated cancer

AOM (Sigma-Aldrich) was dissolved in PBS at 1 mg/ml. Mice were injected intraperitoneally with the AOM solution (12 mg/kg body weight) or vehicle (PBS). Five days after AOM injection, mice were treated by 3 cycles of 2% DSS in the drinking water for 5 days, followed by regular water for 16 days^66^. All mice were euthanized on day 60 and colons were removed for macroscopic inspection and histological analysis. The number of tumors in the size of 1 mm or larger in diameter was counted.

### Quantitative real-time polymerase chain reaction (qRT-PCR)

Total RNA was isolated using the High Pure RNA Isolation Kit (Roche Diagnostics, Basel, Switzerland). cDNA was then synthesized using the High Capacity cDNA Reverse Transcription Kit (Life Technologies, Carlsbad, CA, USA) and 1000 ng of total RNA. qRT-PCR was performed using the StepOne Plus Real-Time PCR system (Life Technologies). The expression of the *Spred2, Cldn1, 2, 4, Muc2* and *Gapdh* gene was analyzed by Taqman gene expression assays (Applied Biosystems). The expression of the *Muc1, Ocln, Cdh1, Tff3, Tjp1* and *Gapdh* gene was analyzed by PrimeTime Mini qPCR assay (Integrated DNA Technologies). The expression level of each gene was normalized to that of the *Gapdh* gene and presented as fold change over the expression of control gene.

### Statistics

Each experiment was repeated at least twice. The two-tailed unpaired Student’s t-test and the One-way analysis of variance (ANOVA) and Tukey’s test were performed using the GraphPad Prism version 5.0b for Mac (San Diego, CA, USA). *p*-values smaller than 0.05 were considered significant.

## Additional Information

**How to cite this article**: Takahashi, S. *et al*. A Novel Role of Spred2 in the Colonic Epithelial Cell Homeostasis and Inflammation. *Sci. Rep*. **6**, 37531; doi: 10.1038/srep37531 (2016).

**Publisher’s note:** Springer Nature remains neutral with regard to jurisdictional claims in published maps and institutional affiliations.

## Supplementary Material

Supplementary Information
