# Supplementary material for: A Novel Role of Spred2 in the Colonic Epithelial Cell Homeostasis and Inflammation
Source: Sci Rep. 2016 Nov 21;6:37531. doi: 10.1038/srep37531 (PMC5116627; doi:10.1038/srep37531)
Supplement: Supplementary Information [file srep37531-s1.docx]

**Supplementary Figures**

A Novel Role of Spred2 in the Colonic Epithelial Cell Homeostasis and Inflammation

Sakuma Takahashi, Teizo Yoshimura, Takahiro Ohkura, Masayoshi Fujisawa, Soichiro Fushimi, Toshihiro Ito, Junya Itakura, Sakiko Hiraoka, Hiroyuki Okada, Kazuhide Yamamoto, and Akihiro Matsukawa

**Supplementary Figure 1. Spred2 knockdown increased the phosphorylation of ERK in Caco-2 cells.** One hundred fifty thousand Caco-2 cells in 1 ml DMEM containing 1% FBS were seeded in 12-well plates. Twenty-four hours after seeding, cells were transfected with siRNA against human Spred2 or non-targeting control siRNA. The expression of Spred2 mRNA (a) or protein (b) was evaluated by qRT-PCR or Western blotting. Western blot signals were quantified by ImageJ software. n = 6. (c) Twenty-four hours after the transfection, cells were stimulated with 100 ng/ml rhEGF. The presence of each protein in the sample at indicated time points was evaluated by Western blotting. (d) Western blot signals were quantitated by ImageJ software. Data are presented as the mean ± SEM. **p < 0.05*, ***p < 0.01*, ****p < 0.001*. n = 6.

**Supplementary Figure 2. Spred2 knockdown increased the migration and proliferation of Caco-2 cells.** (a) The effect of Spred2 knockdown was evaluated by an in vitro scratch assay. Photos were taken at 0 and 48 hours after a scratch was created with a pipette tip. Original scale bar was 200 μm. The front of migrating cells was indicated by solid lines. (b) The migration rates after in vitro wounding of cell monolayer were quantitated by the ImageJ software. Data are presented as the mean ± SEM. n = 6. (c) The proliferation of Caco-2 cells transfected with control siRNA or Spred2 siRNA in the presence or absence of 100 ng/ml rhEGF was examined using a MTT assay. Data is presented as the mean ± SEM. **p < 0.05*, ***p < 0.01*, ****p < 0.001*. n = 6.
